# DETERMINATION OF PHYSICAL ACTIVITY AND SLEEP LEVELS FROM ACTIGRAPH LEAP USING OPEN-SOURCE R PACKAGE

**DOI:** 10.1093/geroni/igae098.3947

**Published:** 2024-12-31

**Authors:** Jethro Raphael Suarez, Chitra Banarjee, Joon-Hyuk Park, Ladda Thiamwong

**Affiliations:** University of Central Florida; University of Central Florida, Orlando, Florida, United States; University of Central Florida, Orlando, Florida, United States; University of Central Florida, Orlando, Florida, United States

## Abstract

Accelerometers are the most common devices used for objectively assessing physical activity (PA) and sleep levels, but cleaning and analysis of the data takes time and requires expertise. Raw Accelerometer Data Analysis (also called GGIR) is an open-source R package that helps researchers process and analyze raw multi-day data from accelerometers and calculates PA and sleep levels. GGIR can process various formats of raw data (e.g.,.csv,.gt3x) from several types of accelerometers but not the raw.csv data from the ActiGraph LEAP device. We aimed to determine PA and sleep levels from LEAP devices by investigating how the raw.csv data file could be manipulated to be compatible with GGIR. We used the pilot data from our NIH-funded study with 24 community-dwelling older adults aged 62 to 91 (mean age = 74.63 ± 7.73 years) who wore the LEAP device for 7 consecutive days. The raw.csv data files were analyzed using a code created in R language that read each raw.csv data file in chunks using the “read.csv” function. We found that manipulation of the column and row structure of the raw.csv file in R resulted in compatibility with GGIR only after removal of the Time Stamps column and matching the header formatting of raw accelerometer.csv files exported from the ActiLife program. Our findings indicated that GGIR can be used to analyze data from LEAP devices and allows for creating a detailed report of PA and sleep levels that benefits both researchers and older adults.

